# Automatic characterization of three cortical neuron types reveals two distinct adaptation mechanisms

**DOI:** 10.1186/1471-2202-12-S1-P119

**Published:** 2011-07-18

**Authors:** Skander Mensi, Richard Naud, Christian Pozzorini, Michael Avermann, Carl CH Petersen, Wulfram Gerstner

**Affiliations:** 1 Brain-Mind Institute, Ecole Polytechnique Federale de Lausanne, 1015 Lausanne EPFL, Switzerland

## 

It has been established over the last 20 years that simplified spiking neuron models are capable of reproducing the variety of firing patterns that have been found in experimental preparations, including delayed spike onset, bursting, strong or weak adaptation, refractoriness, etc. All of these models belong to the family of generalized integrate-and-fire (IF) models, but vary in the way the standard leaky integrate-and-fire model is generalized. Features to upgrade the simple integrate-and-fire include spike after-currents, dynamic threshold, smooth spike initiation and linearized subthreshold currents. Important questions are then: which of these features are needed for basic cortical computation? How many levels of complexity do we have to add to account for relevant features of cortical dynamics? Is the spike-frequency adaptation mediated by moving thresholds or spike-triggered currents?

To answer these question, we developed an efficient method for parameter optimization, that is able to extract some specific features of a neuron from current-clamp experiment. More precisely we are able to estimate the spike-triggered adaptation current that mediates spike-frequency adaptation and the dynamics of the action potential threshold, along with the passive properties of a neuron (i.e. membrane time constant, reverse potential). The method relies on the separation of the parameters affecting the subthreshold voltage and those affecting the firing threshold and its dynamics.

We applied our method to three different neuron classes, Fast Spiking (FS) and non-Fast Spiking (nFS) interneurons and Pyramidal neurons (Pyr). The models we extracted reproduces the excitability type of FS, nFS and Pyr neurons to a remarkable degree of accuracy so that above 90 % of the predictable spikes can be predicted while the difference in subthreshold voltage prediction is less than 1.5 mV. We also find that the adaptation is mediated by different processes in different cell types: mostly by the moving threshold for the Pyr, entirely spike-triggered current for the FS, an equal mix of threshold and current for the nFS.

Finally, we show that the parameters of the adaptation currents and dynamic threshold can be used for an automatic classification of the electrophysiological traces into three well-separated classes, whereas the passive parameters alone do not contain a sufficient amount of information to do so. We observe that the three neuron types have very contrasting threshold dynamics and that efficient classification can be done using only the parameters regulating the dynamics of the threshold.

